# Implementation capital: merging frameworks of implementation outcomes and social capital to support the use of evidence-based practices

**DOI:** 10.1186/s13012-019-0860-z

**Published:** 2019-02-14

**Authors:** Jennifer Watling Neal, Zachary P. Neal

**Affiliations:** Department of Psychology, Michigan State University, 316 W. Physics Rd., East Lansing, 48824 MI USA

**Keywords:** Implementation outcomes, Bridging social capital, Bonding social capital, Social networks, Evidence-based practice

## Abstract

**Background:**

Although there is growing recognition that the implementation of evidence-based practices is a social process, the conceptualization of social capital in implementation frameworks often conflates bonding and bridging social capital. This conflation makes it difficult to concretely operationalize social capital and limits the concept’s utility for explaining implementation outcomes.

**Discussion:**

We propose a new framework of *implementation capital* that merges an existing conceptual framework of implementation outcomes with an existing operational framework of social capital. First, we review a conceptual framework of implementation outcomes, which includes the acceptability, appropriateness, adoption, feasibility, fidelity, cost, penetration, and sustainability of evidence-based practices. Second, we describe an operational framework of social capital that grounds bonding and bridging social capital in the structure of implementers’ social networks. Third, we bring these two frameworks together to create a merged framework of implementation capital that shows how specific aspects of social capital can support specific implementation outcomes. Implementation outcomes of *acceptability*, *appropriateness*, and *adoption* are linked to bonding social capital through mechanisms of trust and norm enforcement, while outcomes of *feasibility* and *fidelity* are linked to bridging social capital through mechanisms of increased access to information and resources. Additionally, setting-level implementation outcomes of *cost*, *penetration*, and *sustainability* are associated with small worldliness at the setting level, which simultaneously optimizes both bonding and bridging social capital in a setting.

**Conclusion:**

The implementation capital framework is helpful because it separates two distinct forms of social capital—bonding and bridging—that are often conflated in the implementation literature, and offers concrete ways to operationalize them by examining the structure of implementers’ social networks and the networks of their settings. This framework offers specific guidance about how individual and setting networks might be shifted to support implementation outcomes.

## Background

The implementation of evidence-based practices (EBPs)^1^ is a social process that requires the people performing the implementation (i.e. implementers) to communicate with and receive social support from multiple actors including other implementers, researchers, developers, support staff, information brokers, and organizational leaders [1–5]. For example, during early stages of implementation, prospective implementers often communicate with other implementers and information brokers to explore and make decisions to adopt EBPs [6–9]. Likewise, during later stages of implementation, implementers often work closely with other implementers, program/practice developers, and support staff to build support and capacity to implement EBPs [2, 5, 10]. Certain aspects of these social relationships facilitate the implementation of new practices, reforms, and EBPs [8, 11–13]. For example, implementers’ adoption and implementation depend on their exposure to social pressure or their access to new information through these relationships. Therefore, frameworks often draw on the role of social relationships, and more specifically the concept of social capital, to understand the implementation of EBPs [13–16].

Despite the widespread use of social capital in implementation frameworks, these frameworks often conflate bonding social capital, which focuses on norm enforcement and trust, with bridging social capital, which focuses on the circulation of resources like information [17–20]. For example, a guide to knowledge translation theory notes that:

Social capital refers to networks with bonding, bridging, and linking capacity to facilitate cooperative, collective, inclusive action and reduce opportunistic behavior. It represents a range of key resources that exist in social relationships, networks, links, connections, associations, customs, and norms [15], p. 32.

Similarly, the Consolidated Framework For Implementation Research (CFIR) includes the concept of social capital as critical to EBP implementation in both the inner and outer settings of organizations, defining it as:

The collective networks of relationships of individuals represent the social capital of the organization. Social capital is one term used to describe the quality and the extent of those relationships and includes dimensions of shared vision and information sharing [14].

These definitions include features of both bonding social capital (e.g., customs, norms, shared vision) and bridging social capital (e.g., access to resources and information). However, they do not attempt to distinguish between these two forms of social capital. Theories of social capital suggest that the distinction between bonding and bridging social capital matters [17–20]. Bonding and bridging social capital involve distinct patterns of social relationships with distinct social consequences for implementation. More specifically, in the context of implementation outcomes, the tightly knit relationships that create bonding social capital lead to norm enforcement and trust that may be important for implementation outcomes related to implementers’ perceptions of EBPs. Likewise, the brokering relationships that create bridging social capital lead to increased access to resources that may be important for implementation outcomes related to implementers’ use of EBPs. Thus, conflating bonding and bridging social capital makes it difficult to concretely operationalize social capital and limits social capital’s utility for explaining implementation outcomes.

To overcome these issues, this debate paper unpacks what social capital is from a structural perspective, then argues that specific forms of social capital can facilitate specific implementation outcomes. By merging a conceptual framework of implementation outcomes [21] with an operational framework of social capital [17, 19], we propose a new framework we call *implementation capital*, which details how implementers’ social networks can support or hinder the implementation of EBPs. We begin by outlining one conceptual framework of implementation outcomes [21]. Next we describe an operational framework of social capital that provides distinct operationalizations of bonding and bridging social capital [17–20]. Finally, we bring these two frameworks together to create a merged framework of implementation capital that highlights how particular aspects of implementers’ social networks can be leveraged to improve specific implementation outcomes.

## A conceptual framework of implementation outcomes

**Fig. 1:**
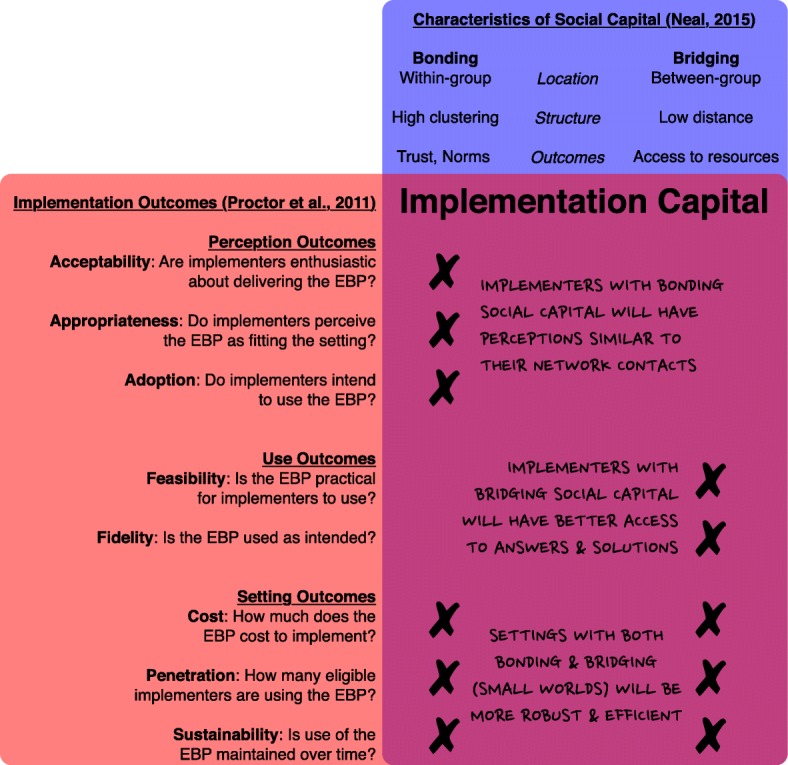
Types of social capital and their link to implementation outcomes

In implementation research, there has been a push to separate more proximal implementation outcomes from distal service outcomes (e.g., service effectiveness, efficiency) or clinical outcomes (e.g., satisfaction, wellbeing [1, 21, 22]). Drawing on elements from existing dissemination and implementation theories including diffusion of innovations [9] and frameworks including RE-AIM [23], Proctor et al. [21] developed a conceptual framework that presents a taxonomy of eight implementation outcomes outlined in Fig. 1.

Three implementation outcomes—*acceptability*, *appropriateness* and *adoption*—focus on implementers’ perceptions of EBPs [21]. Acceptability refers to implementers’ level of satisfaction with and enthusiasm for delivering an EBP. Building on diffusion of innovations theory [9], implementers’ perceptions of the acceptability of an EBP are associated with its complexity (i.e., how easy it is to use?) and relative advantage (i.e., is it better than alternative practices?). Appropriateness refers to whether implementers perceive an EBP as fitting their setting, needs, and experiences, and thus mirrors the concept of compatibility (i.e., does it fit?) [9]. Proctor et al. acknowledge that there is some overlap between the concepts of acceptability and appropriateness, but maintain that it is possible for implementers to perceive an EBP as “appropriate but not acceptable, and vice versa” [21, p. 69]. Finally, adoption refers to whether implementers intend to use or have made an initial decision to use an EBP. This initial adoption decision is also an important part of the dissemination and implementation process described in both diffusion of innovation theory and the RE-AIM framework [9, 23]. Although there is some variation, these outcomes are often of greatest significance early in the implementation process, when implementers are forming opinions about a new EBP.

Two outcomes—*feasibility* and *fidelity*—focus on implementers’ use of EBPs [21]. Feasibility refers to whether an EBP is practical for implementers to use. The feasibility of an EBP is influenced by several characteristics outlined in diffusion of innovations theory including compatibility (i.e., does it fit?), complexity (i.e., is it easy to use?), and trialability (i.e., can it be experimented with on a limited basis; [9]). Fidelity refers to whether an EBP is used by implementers as intended. This maps onto concepts of implementer delivery described as part of the implementation component of the RE-AIM framework [23] as well as Rogers’ [9] discussion of re-invention or the extent to which users of an innovation change it to fit their needs or settings. Again, although there is some variation, these outcomes are often of greatest significance during the implementation process, when implementers attempt to put a new EBP into practice.

Finally, three outcomes—*cost*, *penetration*, and *sustainability*—focus not on individual implementers, but on the settings within which they are engaged in implementation. The cost of implementing an EBP depends not only on the complexity of the practice itself and the complexity of the implementation strategy, but also on the complexity of the setting where it is being implemented [21]. For example, implementation in a stand-alone clinic will involve fewer costs than implementation in a major hospital. Penetration refers to how many eligible implementers within a setting are using the EBP. Penetration is a necessary precursor to the RE-AIM framework’s notion of the reach of an EBP (i.e., the percentage of people within a setting who receive it [23]). Finally, sustainability refers to whether the use of an EBP is maintained over time. This implementation outcome is also described as the maintenance component of the RE-AIM framework and in Rogers’ [9] discussion of how individuals seek reinforcement of their decisions to adopt EBPs.

## An operational framework of social capital

To better understand the relationship between social capital and implementation outcomes, it is necessary to clarify what social capital is. Although the construct of social capital has grown dramatically in popularity, it is over a century old [24, 25]. It has been used to describe specific patterns of relationships and the advantages one gets by having such relationships [17, 18, 20]. As with other forms of capital (e.g., physical capital like tools, human capital like education), we view social capital as a resource that facilitates certain behaviors. But, what kind of resource and what kinds of behaviors?

**Fig. 2:**
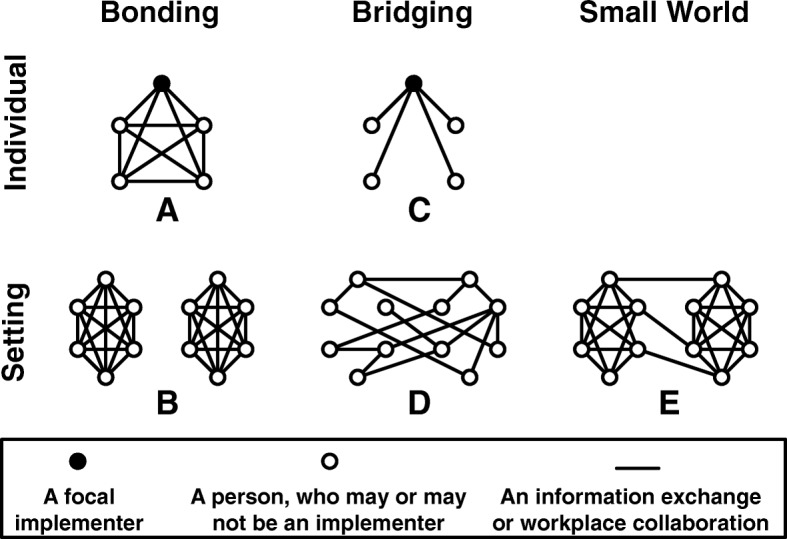
Forms of social capital

Despite significant variation in terminology, the literature on social capital is clear that there are two distinct forms and that each form facilitates a distinct kind of behavior (see Fig. 1). We call one form *bonding social capital*, using the widely known term from Putnam [20], although it is also known as closure [26] and strong ties [18]^2^. An individual has bonding social capital when (or to the extent that) the individuals with whom they interact also interact with one another, which in network science terms is known as *density*. Figure 2a illustrates a focal implementer (the black circle) whose interactions with others (the white circles) are characterized by density, and thus who has bonding social capital. When many individuals in a given setting have bonding social capital, this is reflected in the setting-level network by the presence of dense clusters or groups, which is measured in network science by *clustering* or *transitivity* [19]. Figure 2b illustrates a setting within which each individual (the white circles) has bonding social capital, and thus which is characterized by densely connected groups of other individuals. Whether at the individual or setting level, bonding social capital facilitates a sense of community and reinforces community norms because, as Fig. 2a and b highlight, those with bonding social capital are members of relatively tight-knit closed groups with distinct boundaries. Bonding social capital also allows everyone to trust that others will comply with community norms because the “everyone knows everyone else” pattern of interactions serves as a monitoring system that deters violation of those norms [26]. At the same time, bonding social capital has some disadvantages, most notably, individuals’ lack of access to new ideas from outside the group, which can give rise to groupthink that hampers innovation and reproduces the status quo [27].

Again adopting Putnam’s [20] term, *bridging social capital* represents a second form, which others have called brokerage [17] and weak ties [18]. An individual has bridging social capital when (or to the extent that) their relationships connect them to other individuals and groups that are otherwise not connected to each other, which in network science terms is known as *betweenness*. Figure 2c illustrates an implementer who interacts with others who do not interact with each other; this position of being between others and serving as a gatekeeper for or broker of information gives this implementer bridging social capital. When many individuals in a given setting have bridging social capital, this is reflected in the setting-level network by, in network science terms, *short average distances between nodes* [19]. Figure 2d illustrates a setting within which each individual has bridging social capital, and thus is located only one or two “degrees of separation” from every other individual in the setting. Whether at the individual or setting level, bridging social capital facilitates the rapid and efficient circulation of resources, including information. For example, a piece of information can move from one part of the network to another through a relatively small number of person-to-person interactions. Although bridging social capital offers individuals the advantage of quick access to novel information, it does little to facilitate trust or support because individuals have only one-off interactions with others rather than sustained interactions in groups.

Because bonding and bridging social capital each facilitates different beneficial behaviors, it is ideal to have some of each [17, 19]. However, at the individual level, the patterns of relationships that provide bonding and bridging social capital are opposites; more bonding social capital for an individual means less bridging. However, small world theory from network science has shown that bonding and bridging social capital are not zero-sum at the setting level, where both forms of social capital can simultaneously be high [28]. When a setting is characterized by high levels of clustering, which provides bonding social capital, the formation of even a few of bridging relationships can dramatically reduce the average distance between individuals and thus create bridging social capital [19]. Figure 2e illustrates a setting where each individual enjoys bonding social capital in one of the two dense clusters, but also enjoys rapid access to information because the few bridging links between the clusters ensure each individual is only one or two interactions away from every other individual in the setting. Networks with these characteristics—clustering and short distances—are known as *small world* networks, which have been associated with creativity [29], problem solving [30], and closing the research-practice gap [31].

## A merged framework of implementation capital

Merging the conceptual framework of implementation outcomes [21] with an operational framework of social capital [17–20] has the potential to highlight how implementers’ social networks can be leveraged to improve their implementation of EBPs. Below, we explore how bonding social capital, bridging social capital, and their setting-level combination in small world networks can shape individual perceptions, individual use, and setting-level implementation of EBPs (see Fig. 1).

### Individual perceptions and bonding social capital

Implementation outcomes of acceptability, appropriateness, and adoption focus on implementers’ perceptions of EBPs and thus can be influenced by implementers’ bonding social capital, which reinforces norms, establishes trust, and cultivates a sense of community [17, 19, 26]. When bonding social capital is high, an implementer is integrated in a dense social network of other individuals, who collectively share norms about how things should be done, and whom the implementer is likely to trust. These shared norms and trust mean that the implementer’s perceptions of an EBP will likely be influenced by, and come to resemble those of, others in their network. As a result, bonding social capital can be a double-edged sword for perceptual implementation outcomes. An implementer with significant bonding social capital where others in their network are enthusiastic about an EBP will also likely find the EBP acceptable and appropriate. In contrast, if others in their network lack enthusiasm for an EBP, the implementer will likely find the EBP unacceptable and inappropriate. Diffusion of innovations theory also suggests that a prospective implementer’s adoption of an EBP depends on its adoption by others, and especially by trusted others such as those in one’s own social network [9]. When a prospective implementer has bonding social capital and others in their network have decided to adopt an EBP, the prospective implementer is more likely to also adopt it. However, if others in their network have not adopted an EBP, the prospective implementer is likely to also not adopt it.

Although outcomes of acceptability and appropriateness are often most relevant in the early stages of implementation, they remain relevant in later stages as implementers update and reevaluate their perceptions. For example, implementers may find hard-to-use EBPs less acceptable after attempting to implement them for a while or may find that an EBP is less appropriate than initially expected after putting it into practice in a specific setting. However, bonding social capital can also be relevant for later-stage outcomes of acceptability and appropriateness. Membership in a dense social network—the hallmark of bonding social capital—provides an implementer with a source of support while confronting the inevitable challenges of EBP implementation. Even if others in the implementer’s network are also experiencing challenges, the mere fact that the network establishes a community struggling with implementation together can mitigate declines in acceptability and appropriateness over time.

### Individual use and bridging social capital

Implementation outcomes of feasibility and fidelity focus on implementers’ use of EBPs and thus can be influenced by implementers’ bridging social capital, which facilitates their rapid access to helpful information about how to implement EBPs [17, 19, 26]. Having bridging social capital means that an implementer’s network contacts connect them directly or indirectly with a broad range of sources of information, including those in other parts of their setting (e.g., other teams, departments, or roles) and those outside the setting. Implementers with bridging social capital therefore have access a range of potentially novel (i.e., as opposed to redundant) ideas about how to address implementation challenges when they arise. For example, an implementer who only interacts with colleagues on the same tight-knit team and thus has limited bridging social capital might struggle to find ways to adapt a new EBP to client needs because these colleagues are struggling with the same issues, thus limiting the EBP’s feasibility. In contrast, an implementer who occasionally interacts with others in different roles or departments within their setting, or with an implementer in another state, and thus has high bridging social capital has an opportunity to find ways to make a challenging EBP more feasible by “thinking outside the box” and importing new ideas. Bridging social capital may be particularly important when it links implementers directly or indirectly to an EBP’s development team or other experts in its implementation. Such bridging links mean that implementers can get quicker and more accurate answers to questions about how an EBP should be delivered, thereby boosting the fidelity with which they implement the EBP. Although bridging links do not necessarily connect an implementer with an individual or organization that has relevant information [7], they do connect an implementer with a diverse range of information, which is nonetheless associated with improved problem-solving [32, 33].

### Settings and small worlds

Unlike other implementation outcomes that are focused on individual implementers’ perceptions and use of EBPs, outcomes of cost, penetration, and sustainability focus on the entire setting where implementation occurs. Thus rather than focusing on individual-level forms of social capital (i.e., bonding and bridging), it is more helpful to focus on the structural phenomenon of small worlds, which represents the optimal combination of bonding and bridging social capital at the setting level [19, 28]. A setting will be structured as a small world when participants in that setting (including both implementers and non-implementers) have bonding social capital (e.g., within department-, team-, or role-based clusters), but where a few participants have within-setting ties that bridge across these clusters. However, unlike the implementation outcomes associated with bonding or bridging social capital, we expect that each setting outcome is associated with small world networks for slightly different reasons.

First, although implementation cost can include exogenous factors such as the actual time and financial costs of using an EBP, it can also be impacted by the complexity of the implementation setting itself [21]. Implementation cost will be high in settings characterized primarily by bonding social capital, where participants are in sets of densely connected but isolated groups (see Fig. 2b), because implementation will involve significant redundancies (e.g., multiple trainings must be conducted in each isolated team). Cost will also be high in settings characterized primarily by bridging, where participants lack a clear team structure or hierarchy (see Fig. 2d), because implementation efforts will be difficult to coordinate. In contrast, a setting structured as a small world (see Fig. 2e) balances these competing pressures because the bonding social capital it provides allows coordination within teams, while the bridging social capital it provides allow economies of scale in the delivery of training.

Second, penetration of an EBP’s implementation requires the penetration of two distinct phenomena: implementers’ knowledge about the EBP and implementers’ use of the EBP [9, 34, 35]. A setting structured as a small world is ideal for achieving both. Penetration of implementers’ knowledge about an EBP (i.e., dissemination) is facilitated by the bridging social capital of a small world network, which makes it possible for information to reach all setting participants efficiently. The bridging ties ensure that information about an EBP (e.g., its existence, its basic practices and tools) can spread throughout the entire setting via a relatively small number of intermediaries, which helps transmit the information both quickly and without distortion. Conversely, penetration of implementers’ use of an EBP (i.e., implementation) is facilitated by the bonding social capital of a small world network. The bonding ties form clusters in the setting’s network, which can serve as communities of practice that encourage continued use by providing reinforcement and support to implementers during the implementation process.

Finally, a key challenge to the sustainability of EBP implementation is turnover, which can be high in many social service settings [36]. Frequently, settings are structured hierarchically, where the departure of a single person fragments the network of communication, hampering both trust and information sharing, as well as leading to ambiguity, confusion, and hampered implementation outcomes. In contrast, a setting structured as a small world mitigates the impact of turnover because the departure of one person from the network has little impact on the network’s overall structure [37]. For example, the departure of any one or two individuals from Fig. 2e does not substantially reduce the network’s clustering and setting’s ability to facilitate trust, or the network’s compactness and setting’s ability to facilitate information sharing. Such a setting is resilient to the challenges that accompany turnover and thus is more likely to facilitate the sustained implementation of an EBP when turnover is inevitable.

## Conclusion

Although there is growing recognition that the implementation of EBPs is a social process [1–5], the conceptualization of social capital in implementation frameworks remains vague [14, 15]. In this paper, we propose a new framework of implementation capital that merges a conceptual framework of implementation outcomes [21] with an operational framework of social capital [17, 19]. The implementation capital framework offers several advantages over current discussions of social capital in the context of implementation science. First, it is helpful because it separates two distinct forms of social capital—bonding and bridging—that are often conflated in the implementation literature, and offers concrete ways to operationalize them by examining the structure of implementers’ social networks and the networks of their settings.

Second, by offering clarity around types of social capital, the implementation capital framework implies specific hypotheses regarding how social capital is associated with implementation outcomes. Accordingly, it answers a call for theory-building research that involves “treating [implementation outcomes] as dependent variables, in order to identify correlates and predictors of their attainment” [21], p. 73. For example, while implementation outcomes of acceptability, appropriateness, and adoption may be linked to bonding social capital through mechanisms of trust and norm enforcement, other outcomes of feasibility and fidelity may be linked to bridging social capital through mechanisms of increased access to information and resources. Additionally, setting-level outcomes of cost, penetration, and sustainability may be associated with small world network configurations that provide an optimal combination of bonding and bridging social capital.

Finally, if these hypotheses are supported, the framework can offer specific guidance about how individual and setting networks might be shifted to support implementation outcomes. For example, professional learning communities (PLCs)—“a group of people sharing and critically interrogating their practice in an ongoing way” [38, p. 223]—have been identified as a strategy for increasing implementers’ social capital and their implementation of EBPs [39–41]. The implementation capital framework suggests that PLCs might be particularly well-suited to promoting bonding social capital because they bring a consistent group together to share and discuss, and thus that PLCs are a pathway toward implementation outcomes of acceptability, appropriateness, and adoption. At the same time, others have suggested promoting EBP implementation by finding ways to link implementers to people and organizations outside their immediate settings (i.e., building bridging social capital) who can provide advice and expertise [7, 8, 30, 31, 42]. For example, Palinkas et al. [8] describe the formation of community development teams to build mental health providers’ information and advice networks across counties, while Bunger et al. [39] describe building clinicians’ links to training experts. If strategies like PLCs can build bonding social capital among individual implementers, and strategies like building new ties to outside individuals or experts can build bridging social capital among individual implementers, then the settings in which these implementers are embedded will have the characteristics of a small world and will facilitate setting-level outcomes of cost, penetration, and sustainability.

Although the implementation capital framework offers several advantages over existing syntheses of social capital and implementation science, it also raises several questions that identify directions for future research. First, what role do factors like political pressures or resource availability, which are known to impact implementation outcomes but are not directly linked to implementers’ (or implementers’ settings’) social networks, fit in? The implementation capital framework focuses specifically on the impact of social factors, and specifically social structural factors, which do not operate in a vacuum and are likely moderated by other forces. For example, if there are no resources available to implement a particular EBP, then even an optimal combination of bonding and bridging social capital may have little effect on implementation outcomes. Similarly, if there are strong political incentives to implement (or not implement) a particular EBP, they may reinforce or mitigate the impact of implementers’ social capital. Accordingly, the implementation capital framework is not a replacement for other ways of understanding implementation outcomes, but instead can be used alongside other approaches to more explicitly and concretely examining the effects of social relationships.

Second, how can implementers’ social capital be measured? The tools of social network analysis provide many options for measuring social capital at both the individual [43, 44] and setting [19] levels. Despite their differences, these techniques all involve the measurement of individuals’ relationships, typically via a brief survey [45], where collecting data about the relevant types of relationships is critical [46]. In the context of implementation outcomes, interactions of information exchange and workplace collaboration are likely among the most important for social capital. Yet, this requires empirical verification. In large, complex, or fast-changing settings, collection of implementers’ network data may be impractical, especially when much of implementers’ time is devoted to the implementation itself. In such cases, caution should be exercised using a non-network assessment of social capital [47], for example which tend to focus on measuring the consequences of having social capital (e.g., trust) and often do not distinguish bonding from bridging forms. However, the implementation capital framework can still offer heuristic guidelines for improving implementation outcomes through social capital, for example by recommending activities that build communities of practice (i.e., bonding social capital) while at the same time ensuring that implementers also have some interactions with those outside their settings (i.e., bridging social capital).

Third, why do implementers (and their settings) have the networks and social capital that they do? Understanding the origins of specific network structures is a central concern for the field of social network analysis. While common demographic characteristics like gender are important [48], some factors unique to the context of implementation may also matter. For example, an EBP can arrive in a setting via a demand-side *pull* process in which implementers locate an EBP believed to meet their needs, or via a supply-side *push* process in which researchers encourage and facilitate implementers’ use of an EBP [49, 50]. Although the implementation capital framework is agnostic about the origins of an EBP, its arrival process may nonetheless be associated with implementers’ social capital. Specifically, when a researcher pushes an EBP out to a setting, this forges a bridging link between the practice setting and research world that enhances implementers’ bridging social capital. More generally, however, understanding the forces that shape implementers’ networks and social capital is critical for using the implementation capital framework to purposefully promote networks that will improve implementation outcomes.

## Abbreviations

CFIR: Consolidated framework for implementation research
EBP: Evidence-based practice
PLCs: Professional learning communities
RE-AIM: Reach, efficacy or effectiveness, adoption, implementation, and maintenance

## Footnotes

1 There are many names for the things that implementation science studies the implementation of: evidence-based practices (EBPs), empirically supported treatments (ESTs), innovations, and interventions. For simplicity, in this paper, we use EBPs as a general term intended to encompass all practices, treatments, innovations, and interventions that have an evidence base. Our definition is inclusive of EBPs that are implemented by single individuals as well as those implemented by groups or teams of individuals in collaboration.

2 We use Putnam’s [20] terms here for convenience and because these terms have been employed in implementation science [14, 15], but this choice does not imply our agreement with other nuances of his theory, which is unrelated to our central argument about implementation outcomes.
